# Cooperation among Microorganisms

**DOI:** 10.1371/journal.pbio.0040299

**Published:** 2006-09-12

**Authors:** Ned S Wingreen, Simon A Levin

**Affiliations:** Princeton University, United States of America

## Abstract

Understanding cooperation among microorganisms presents conceptual and mathematical challenges at the interface of evolutionary biology and the theory of emergent properties of independent agents, two of the most exciting areas in modern mathematical biology.

One of the organizing principles of life on Earth is that cells cooperate. This is evident in the case of multicellular organisms, from nematodes to humans, but it also appears to apply widely among single-celled organisms such as bacteria, fungi, and amoeba. In many cases, the label “single-celled” applies to only part of the life cycle of these organisms. For example, the model amoeba *Dictyostelium discodium* is single-celled under conditions of nutritional abundance, but upon starvation, it communicates to form aggregates that subsequently pass through multicellular stages of slug and fruiting body. Indeed, in light of recent discoveries of communication among bacteria and the importance and prevalence of bacterial biofilms, “single-celled” may turn out to be a misnomer even for these organisms. Here we highlight some of the better-studied examples of cooperation among microorganisms and attempt to identify some of the important questions in this emerging field. Understanding cooperation among microorganisms presents conceptual and mathematical challenges at the interface of evolutionary biology and the theory of emergent properties of independent agents, two of the most exciting areas in modern mathematical biology.

Most of the best-studied cases of cooperation among microorganisms concern intraspecies cooperation. An example of this is quorum sensing among bacteria, in which cells produce, secrete, and detect small molecules, called autoinducers. At high enough autoinducer concentrations (high cell densities), the bacteria enter a new mode of existence characterized by expression of genes associated with collective behaviors that are best carried out in concerted fashion by many cells [1]. These behaviors include the formation of protective biofilms, the expression of virulence factors to attack a host, the production of light, the establishment of competence to exchange DNA (a bacterial form of sexual recombination), and many others. The signaling pathway for one of the better- studied quorum-sensing circuits, that of *Vibrio cholerae*, the human pathogen, is shown in Figure 1.

**Figure 1:**
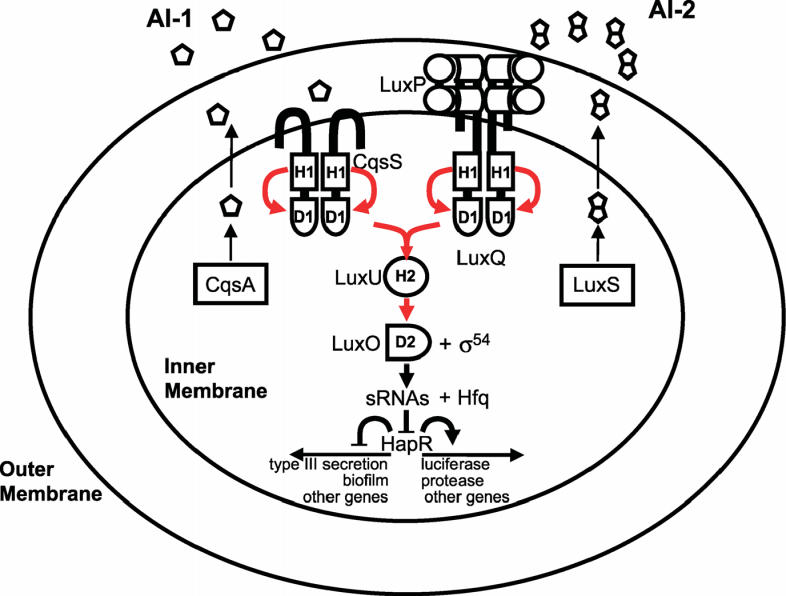
Quorum-Sensing Circuit of the Bacterial Pathogen *Vibrio cholerae* Red arrows indicate phosphoryl-group transfer [1]. (Figure: Matthew B. Neiditch, Princeton University, Princeton, New Jersey, United States)

Another well-studied example of intraspecies cooperation concerns the cyanobacterium *Anabaena*, which grows in long chains, in which approximately one cell out of ten differentiates into a heterocyst that provides fixed nitrogen for the neighboring cells (Figure 2) [2]. *Dictyostelium* is probably the most-studied model for cooperation among eukaryotic microorganisms, but even in the nonmotile eukaryote *Saccharomyces cerevisiae*, hyphal growth (that is, filamentous growth) can be viewed as a cooperative mechanism for foraging.

**Figure 2:**
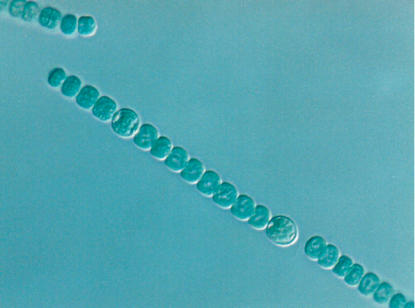
Heterocyst Differentiation in *Anabaena* sp. *UTCC-426* (Photograph: Mary Olaveson, University of Toronto Scarborough, Toronto, Ontario, Canada)

Cooperation between different microorganism species is much less understood, or studied, partially for practical reasons, but also because the ubiquity of communication among microorganisms has only recently been appreciated. Nevertheless, it has been clear for many years that bacteria form biofilms on many surfaces (including human teeth, artificial joints, and organs, as well as on the surfaces and in the roots of plants, including crops) that consist of large consortia of different organisms. Moreover, it is clear that, far from being a case of pure Darwinian competition, interactions among these species and with eukaryotic hosts may be mutually beneficial. A recent case in point is the discovery of a mutualistic interaction of four bacterial species with the tomato plant (M. del Gallo, personal communication). Rather than competing, the four species coexist and strongly promote plant growth by fixing nitrogen, providing growth hormones, and preventing hostile bacterial species from growing. Tooth biofilms have been shown to consist of stable consortia of hundreds of distinct species, and bacterial mats are believed to consist of even larger numbers of species, in dynamic equilibrium among themselves, and with multiple bacterial viruses. Interest in bacterial cooperation has been spurred by the discovery that one of the autoinducers, named AI-2 (a furanone), is produced by a wide variety of bacteria, including most known human pathogens, and it may be one of a class of universal interspecies communication molecules [3,4].

These examples highlight the range of behaviors that could be termed “cooperation.” Cooperative behaviors include complex social interactions such as division of labor and mutualism in providing shelter, foraging, reproduction, and dispersal [5]. The examples also highlight the importance of communication in adjusting group behavior to environmental circumstances and population density. Cooperation also has its discontents, and there is growing interest in the role and fate of “cheaters” among microorganisms. There is some evidence as well for “police,” particularly in the context of bacterial-host interactions, in which host systems favor the growth of symbiotic bacteria but discourage growth of noncooperative, but otherwise identical, cells [6,7]. For a recent review of communication in bacteria that highlights these issues, see [8].

Understanding how cooperation arose and is maintained, particularly among large numbers of species, presents a challenge for practitioners of both molecular biology and evolutionary biology, as well as for theorists. Is cooperation best understood as the convergence of the immediate self-interest of multiple parties? Or can evolution lead to stable cases of short-term altruistic behavior, providing long-term benefit for all? These questions have been central in evolutionary biology since the time of Darwin, who regarded apparently altruistic behavior as a challenge for his theory. Especially puzzling was the extreme levels of cooperation and altruism, termed eusociality, in the haplodiploid insects and termites.

J. B. S. Haldane elucidated a fundamental principle underlying apparent altruistic behavior when he said that he would lay down his life to save two brothers or eight cousins, reflecting the one-half and one-eighth of his genes he shared with each, respectively. William D. Hamilton formalized these notions in his theory of kin selection, pointing out that the enhanced genetic relatedness of haplodiploid sisters, who share three-quarters of their genes, facilitates “altruism” in the haplodiploid species. Subsequent work has shown that kin selection can also work effectively under conditions of low relatedness and, furthermore, is not even necessary for cooperative behavior to arise. Cooperation can similarly be facilitated among unrelated individuals, for example, when the spatial range of interactions is restricted. Kin selection may play a role when limited spatial range is involved, but it is not essential [9]. On the other hand, a limited range of spatial interactions is no guarantee of cooperation; it can just as well lead to spite and selfish behavior, as in the production of allelopathic substances in microorganisms and plants [10]. For reviews of the selective mechanisms leading to cooperation and altruism, see [11–13].

The challenges in understanding cooperation and how it becomes reinforced over evolutionary time to produce stable mutualisms and even multicellularity is at the core of understanding biology. It is key to understanding how complexity arose evolutionarily, how organisms band together and profit from collective decision making, and how populations of diverse organisms interact to produce self-reinforcing networks of mutual benefit. It is also key to understanding the maintenance of ecological communities and patterns of nutrient cycling. The mathematical approaches of the past provide a foundation, but new mathematical techniques drawn from such diverse subjects as dynamical game theory and spatial stochastic processes will be needed to lay bare the essential truths. Considerable progress has been made in the past few years in developing the relevant mathematics, and we are at the threshold of dramatic advances in our understanding of cooperative behavior, one of the central and fundamental issues in biology.
